# Evaluation of Global Immune Biomarkers to Predict Infections in Lung Transplant Recipients

**DOI:** 10.1111/ctr.70654

**Published:** 2026-08-17

**Authors:** Quoc Nguyen, Michael J. Lydeamore, Adina Dessauer, Gregory I. Snell, Glen P. Westall, Anton Y. Peleg, Bradley J. Gardiner

**Affiliations:** ^1^ Department of Econometrics and Business Statistics Monash University Melbourne Victoria Australia; ^2^ Department of Infectious Diseases Alfred Health Melbourne Victoria Australia; ^3^ Lung Transplant Service, Department of Respiratory Medicine Alfred Health Melbourne Victoria Australia; ^4^ School of Translational Medicine Monash University Melbourne Victoria Australia; ^5^ Centre to Impact AMR Monash University Clayton Victoria Australia; ^6^ Infection Program, Monash Biomedicine Discovery Institute, Department of Microbiology Monash University Clayton Victoria Australia

**Keywords:** immune monitoring, infection, lung transplant, lymphocyte count

## Abstract

**Background:**

Immune biomarkers could assess levels of immunosuppression and predict infections in transplant recipients. We aimed to evaluate the absolute lymphocyte count (ALC), absolute neutrophil count (ANC), Quantiferon‐Monitor and the mitogen component of the Quantiferon‐CMV assay alone and combined to predict serious opportunistic infections (SOI) in lung transplant recipients.

**Methods:**

Patients were prospectively recruited from 2015–17, with blood collected at 3, 6 and 12 months post‐transplant. Infections were classified as SOI if requiring hospitalization and caused by an opportunistic pathogen. Logistic regression was used to calculate odds ratios (OR).

**Results:**

Within 18 months post‐transplant, 35/80 patients experienced 46 SOI's. Low ALCs were associated with SOI between 3–6 (median 1.4 vs. 1.0×1000 cells/µL, OR 5.38 per unit decrease, 95% CI 1.38–21.02, *p* = 0.02) and 6–12 (1.3 vs. 0.8×1000 cells/µL, OR 3.48, 95% CI 1.20–10.1, *p* = 0.02) months. Mitogen values were significantly lower with SOI between 3–6 months (5.8 vs. 0.3 IU/mL, OR 0.78, 95% CI 0.63–0.97, *p* = 0.02). Area under the ROC curve for all biomarkers combined were modestly higher compared to ALC alone. Negative predictive values were high.

**Conclusions:**

Immune biomarkers have the potential to measure net immunosuppression and identify transplant recipients at higher risk for SOI. ALC was the most useful single test although incorporating 3 other biomarkers modestly improved predictions. Negative predictive values were high indicating that patients with high ALC values were unlikely to experience SOI. While this approach could inform decision‐making regarding immunosuppression dosing, intensity of monitoring and antimicrobial prophylaxis, further research is required before clinical implementation.

**Abbreviations**
ALC: absolute lymphocyte count
ANC: absolute neutrophil count
BAL: bronchoalveolar lavage
CI: confidence interval
CMV: cytomegalovirus
D: donor
EBV: Epstein‐Barr virus
HR: hazard ratio
HSV: herpes simplex virus
IQR: interquartile range
IU: international units
LTR: lung transplant recipients
OR: odds ratio
QF‐CMV: Quantiferon‐Cytomegalovirus
QFM: Quantiferon‐Monitor
R: recipient
SOI: serious opportunistic infection
SOT: solid organ transplantation
VZV: varicella zoster virus.

## 1 Background

Lung transplantation offers the potential for a new lease on life for patients with end‐stage lung disease. Intensive lifelong immunosuppression, necessary to prevent rejection, increases the risk of serious infections which contribute to substantial morbidity and mortality. [1] Despite many advances in transplant medicine, management of immunosuppression remains mostly protocol‐driven and reactive, guided by time post‐transplant, clinical factors such as age, weight, comorbidities, donor‐recipient match, and calcineurin or mammalian target of rapamycin inhibitor levels. [2] Precise targets that achieve appropriate immunosuppression for individual patients are difficult to define. [3, 4, 5]

Several novel immune biomarkers are now available and have been explored to measure immunosuppression and predict infections. [1, 6] These could help inform individualized dosing, minimizing risk of both inadequate immunosuppression resulting in rejection and excessive immunosuppression leading to infections, malignancies and medication toxicities. Some of these have been targeted towards specific pathogens such as cytomegalovirus (CMV), the most common and significant infection in transplant recipients. [7, 8] Others are pathogen‐agnostic ‘global’ immune biomarkers that can predict infections more generally. The Quantiferon‐Monitor (QFM, Qiagen, Germantown, MD, USA) is novel whole blood immune function assay that measures plasma interferon‐γ levels following stimulation with innate and adaptive immune antigens. Emerging data suggest that low values are associated with higher degree immunosuppression and could identify patients at higher risk of serious infections. [6, 9, 10] Low absolute lymphocyte counts (ALC) have been independently associated with an increased risk for infections, particularly CMV, in a growing number of studies. [8, 11, 12, 13, 14] The Quantiferon‐CMV assay (QF‐CMV, Qiagen Inc, Germantown, MD, USA) includes a mitogen control tube which measures response to phytohemagglutinin, a non‐specific immune antigen. Patients with indeterminate results due to low mitogen values appear to be at higher risk for CMV infection. [15, 16] Finally, low absolute neutrophil counts (ANC) are a well‐recognized risk factor for infections, particularly bacterial sepsis and invasive fungal infections. [17, 18, 19, 20] While promising, data on each individual biomarker is heterogeneous making it difficult to apply these assays clinically to evaluate the risk of opportunistic infections in lung transplant recipients.

We previously explored QFM in lung transplant recipients and found that prednisolone dose was strongly inversely associated with QFM level (0.1 mg/kg increase correlating with 88 IU/mL QFM decrease, 95% CI 61–114, *p* < 0.001). [9] Very early post‐transplant, all patients were highly immunosuppressed, and infections were more likely to be related to surgical, donor and nosocomial factors rather the net state of immunosuppression. Patients with QFM values <10 and <60 IU/mL were more likely to develop serious opportunistic infections between 3–6 months (HR 6.38, 95% CI 1.37–29.66, *p* = 0.02) and 6–12 months (HR 3.25, 95% CI 1.11–9.49, *p* = 0.03) post‐transplant respectively. There were no clear relationships between QFM results and rejection. We hypothesized that including additional biomarkers could increase overall predictive accuracy. The aims of this study were therefore to evaluate four global immune biomarkers (ALC, ANC, QFM and the mitogen component of the QF‐CMV assay) alone and in combination for the prediction of serious opportunistic infections in lung transplant recipients.

## 2 Methods

### 2.1 Study Design & Population

Patients were prospectively recruited from the Alfred Hospital in Melbourne, Australia from August 2015 to July 2017. The study was approved by the Alfred Health Ethics Committee with written informed consent obtained from all participants. Patients were excluded if they were <18 years old, unable to consent, previously received a transplant, or if long‐term follow‐up was planned elsewhere. Blood was collected at 3, 6 and 12 months following transplant. Additional samples were also available from 2 and 6 weeks post‐transplant but were not included in the analysis due to the very low event rates in this period. Clinical data were obtained from hospital medical records. All patients underwent surveillance bronchoscopy with bronchoalveolar lavage (BAL) at 2, 6, 12, 24 and 52 weeks post‐transplant and when clinically indicated. BAL fluid was sent for bacterial, mycobacterial and fungal cultures, CMV viral load testing, cytology and other diagnostics as indicated. Transbronchial lung biopsies were collected at all bronchoscopies and sent for histopathologic evaluation. Lung function testing was performed at least monthly coinciding with outpatient clinic attendance.

### 2.2 Laboratory Methods

The QFM and QF‐CMV assays were performed according to the manufacturer's instructions in our standard diagnostic laboratory. For QFM, 1 mL of blood was collected in a dedicated tube, a LyoSphere containing anti‐CD3 (T‐cell stimulant) and R848 (a Toll‐like receptor 7 ligand) was added within 8 h and the sample incubated for 16‐24 h at 37°C, then centrifuged to obtain plasma. For QF‐CMV, 1 mL of whole blood was collected in each of the 3 tubes (nil control, mitogen control and CMV antigen) then incubated at 37°C for 16–24 h. An enzyme‐linked immunosorbent assay for interferon‐γ was performed on all tubes. QFM values were reported on a continuous scale from 0–1000 IU/mL. QF‐CMV was classified as positive (suggesting an adequate CMV‐specific immune response, also termed detected/reactive) if a level ≥0.2 IU/mL was found in the CMV tube, negative (non‐reactive/not detected) if ≥0.5 IU/mL in the mitogen tube but <0.2 IU/mL in the CMV tube, and indeterminate if the mitogen tube was <0.5 IU/mL and the CMV <0.2 IU/mL. Mitogen values were also reported on a continuous scale from 0–10 IU/mL. QFM and mitogen results were not available to treating clinicians in real‐time.

### 2.3 Immunosuppression, Rejection and Antimicrobial Prophylaxis

Standard maintenance immunosuppression included a calcineurin inhibitor (typically tacrolimus), an antiproliferative drug (typically azathioprine) and prednisolone. Basiliximab induction was used in patients at high risk of renal dysfunction. Acute cellular rejection was diagnosed on transbronchial biopsy according to the International Society for Heart and Lung Transplantation (ISHLT) scoring system. [21] Patients with rejection more than grade A2 were treated with intravenous methylprednisolone 15 mg/kg daily for 3 days, as were patients with clinically suspected acute rejection in the absence of a confirmatory biopsy.

Perioperative antibacterial prophylaxis typically included an antipseudomonal beta‐lactam (e.g., piperacillin‐tazobactam) for 10–14 days, modified according to donor/recipient microbiology. *Pneumocystis jirovecii* prophylaxis with trimethoprim‐sulfamethoxazole was commenced around 2 weeks post‐transplant and continued indefinitely. Routine antifungal prophylaxis was not used, instead patients with mold identified on pre‐ or post‐transplant microbiologic testing received a mold‐active azole for 3 months. Otherwise, routine oral nystatin was used to prevent oral candidiasis. All CMV seropositive recipients (R+) and seronegative recipients receiving an organ from a CMV seropositive donor (D+/R‐) received primary antiviral prophylaxis for 5 or 11 months respectively with IV ganciclovir initially then oral valganciclovir 450 mg twice daily once tolerated, renally dose‐adjusted and generally discontinued a month before a planned bronchoscopy. D+/R‐ patients also received 7 doses of CMV immunoglobulin. Donor and recipient CMV seronegative patients received valaciclovir for 3 months.

### 2.4 Infection Outcomes

Clinical records of all cases were carefully reviewed by a panel of experienced infectious diseases/lung transplant physicians blinded to biomarker results to determine if they experienced infection within the first 18 months post‐transplant. The primary outcome was serious opportunistic infections (SOI), defined as an infection related to immunosuppression that does not typically affect healthy individuals and resulted in new or extended hospitalization (Table S1). These included pathogens such as *Nocardia* spp., cytomegalovirus (CMV), Epstein‐Barr virus (EBV) including post‐transplant lymphoproliferative disease, disseminated varicella zoster virus (VZV) or herpes simplex virus (HSV), human herpesvirus 6/7/8, non‐tuberculous mycobacteria, disseminated tuberculosis, fungal infections from *Aspergillus*, other molds, *Cryptococcus*, *Pneumocystis jirovecii*, and invasive or esophageal candidiasis. Episodes of disseminated pneumococcal infection, *Stenotrophomonas* pneumonia, bacteremia due to vancomycin‐resistant *Enterococcus*, oral/vaginal candidiasis, mold colonization and asymptomatic isolated detectable but non‐quantifiable CMV viremia in either blood or BAL were not classified as opportunistic infections. Definitions were adapted from previous studies and modified to suit our patient population and local diagnostic testing. [9, 22, 23, 24, 25, 26, 27, 28, 29]

### 2.5 Statistical Analysis

Categorical data were reported as counts and percentages, continuous data as means ± standard deviations if normally distributed and medians with interquartile range if non‐normal. Preliminary analyses were performed with basic comparative statistical methods. To analyze infection outcomes, follow‐up was split into three time periods (3–6, 6–12 and 12–18 months post‐transplant), with events occurring within each period analyzed based on biomarker results from the beginning of the period. Univariable and multivariable odds ratios (OR) with 95% confidence intervals (CI) were calculated using logistic regression. OR are presented per unit decrease in each biomarker value. Receiver‐operating characteristic (ROC) curves were constructed to determine optimal cutoffs and calculate the accuracy of biomarkers for predicting SOI. Models were compared using the concordance (c) statistic or area under the ROC curve, Akaike information criterion (AIC). Kaplan‐Meier survival curves representing time to infection were generated. Hazard ratios (HR) were calculated with Cox proportional hazards models. ANC, ALC and mitogen values were considered missing if no results were available within 10 days of QFM testing. Patients were followed up until death or administrative censoring, which occurred at the earliest of loss to follow‐up, end of the study period or 18 months post‐transplant. P‐values of <0.05 were considered statistically significant. Analyses were performed using the R statistical software platform version 4.4.1 (RStudio version 2024.04.2+764).

## 3 Results

### 3.1 Cohort Description

Eighty lung transplant recipients were originally recruited (Figure S1). Median age was 61 years, 48 (60%) were male, 69 (86%) underwent bilateral lung transplantation and 40 (50%) received basiliximab induction (Table S2). The most common indications for transplant were chronic obstructive pulmonary disease (34/80, 43%) and pulmonary fibrosis (24/80, 30%). There was no loss to follow‐up although a few patients did not have all biomarkers tested at all timepoints (Table S3). One patient died of a cardiac event before any biomarkers could be collected, leaving 79 patients with data available. There were five additional deaths during the first 18 months post‐transplant. This resulted in ANC & ALC values for 70/75 (93%) and QFM & mitogen values for all patients remaining alive at 12 months. One patient underwent re‐transplantation at 7 months. Four patients received thymoglobulin, at a median of 15.2 months post‐transplant. Overall, 25 patients (31%) received high‐dose steroids for rejection.

The trajectories of the biomarkers over time is shown in Figure S2 and Table S3. QFM values were very low early post‐transplant then progressively increased over the year, from a median 3 IU/mL at 2 weeks to 178 IU/mL at 12 months. Mitogen values also rose gradually over the year from a median of 0.3 IU/mL to 8.2 IU/mL, and had the widest variation in results, especially at later timepoints. Lymphocyte counts were relatively stable over time, with median values ranging between 1.1–1.3×1000 cells/µL. ANC was higher early (median 8.5×1000 cells/µL) then progressively decreased to a median of 3.7×1000 cells/µL at 1 year.

### 3.2 Infection Outcomes

In the first 18 months post‐transplant there were 375 infection episodes, 177 of which were classified as serious and 95 as opportunistic infections. Forty‐six episodes in 35 patients met our definition of SOI (Table S4, Figure S3). These predominantly occurred during the first‐year post‐transplant (41 episodes in 34 patients) and were less frequent between 12–18 months (5 episodes in 4 patients). These included 14 episodes of CMV, 14 invasive mold infections, 4 cryptococcal infections and 14 others (Table S5). Median duration of hospitalization per SOI episode was 7 days (IQR 5–15 days). Overall, within 18 months post‐transplant 26 patients experienced a single SOI, 8 had two episodes and one patient had 4 SOIs. Nine episodes of SOI in 7 patients occurred within the first 3 months post‐transplant so were not included in subsequent analyses. The only clinical factor that was significantly associated with SOI was longer duration of initial post‐transplant hospitalization (median 21, IQR 17–23 versus 18, 16–38 days, HR 1.02, 95% CI 1.01–1.03, *p* = 0.002). Patients at risk for CMV due to either previous infection (R+) or donor seropositivity (D+/R‐) were at higher risk of SOI, but this did not reach statistical significance (Table S6).

### 3.3 Immune Biomarkers and Serious Opportunistic Infections

**FIGURE 1:**
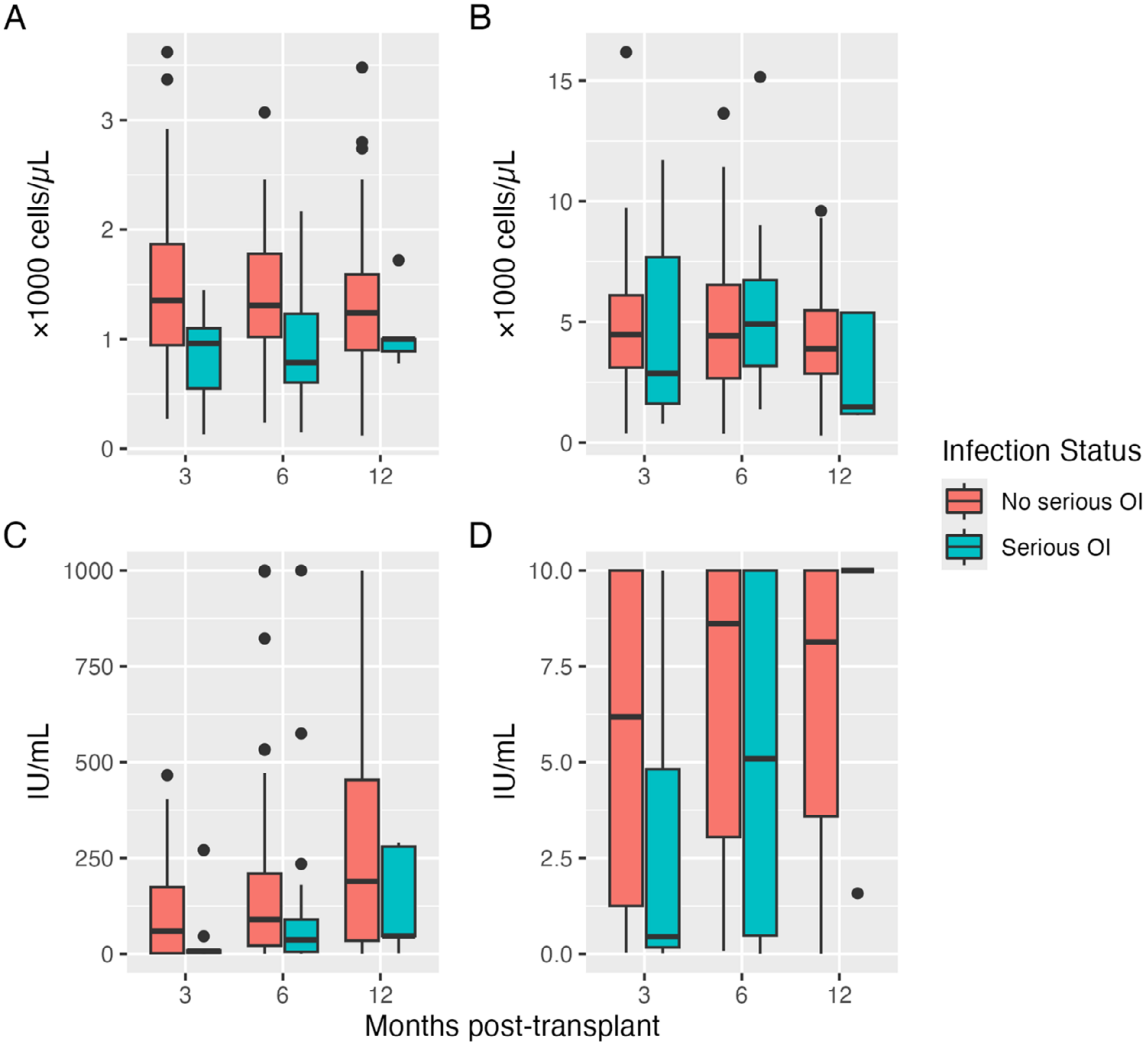
Immune biomarker values at 3, 6 and 12 months post‐transplant in patients who did and did not experience serious opportunistic infections, including (a) absolute lymphocyte count, x1000 cells/µL (b) absolute neutrophil count, x1000 cells/µL (c) Quantiferon‐Monitor, IU/mL and (d) mitogen values, IU/mL. OI, opportunistic infection. ANC, absolute neutrophil count. ALC, absolute lymphocyte count. QFM, Quantiferon Monitor.

A comparison of biomarker values in patients who did and did not experience SOI is shown in Figure 1. Low ALCs were significantly associated with SOI between 3–6 (median 1.4 vs. 1.0×1000 cells/µL, OR 5.38 per unit decrease, 95% CI 1.38–21.02, *p* = 0.02) and 6–12 (1.3 vs. 0.8×1000 cells/µL, unadjusted OR 3.48, 95% CI 1.20–10.1, *p* = 0.02) months (Tables 1 & S7). Mitogen values were also significantly lower in patients with SOI between 3–6 months (median 5.8 vs. 0.3 IU/mL, unadjusted OR 1.28, 95% CI 1.03–1.59, *p* = 0.02). While biomarkers tended to be lower in patients with SOI in other time periods, they did not reach statistical significance.

**TABLE 1:** Comparison of biomarker values in lung transplant recipients who did or did not develop serious opportunistic infections between 3‐6, 6‐12 and 12‐18 months post‐transplant, with univariate odds ratios.

Months post‐transplant	Biomarker	No serious OI Median, IQR	Serious OI Median, IQR	Unadjusted OR^*^ (95% CI)	*p*‐value
3–6	ALC	1.4, 0.9–1.9	1.0, 0.5–1.1	5.38 (1.38–21.02)	0.02
	ANC	4.4, 3.1–6.1	3.7, 1.7–8.1	0.97 (0.77–1.22)	0.81
	Mitogen	5.8, 1.2–10	0.3, 0.2–2.5	1.28 (1.03–1.59)	0.02
	QFM	60, 2–180	7, 3–9	1.09 (0.98–1.20)	0.11
6–12	ALC	1.3, 1–1.8	0.8, 0.7–1.2	3.48 (1.20–10.10)	0.02
	ANC	4.4, 2.7–6.5	5.4, 3.3–6.9	0.95 (0.80–1.12)	0.55
	Mitogen	9, 3.6–10	7.8, 1.1–10	1.07 (0.93–1.22)	0.36
	QFM	87, 14–262	41, 6–90	1.01 (0.98–1.03)	0.46
12–18	ALC	1.3, 0.9–1.6	0.9, 0.9–1.2	2.02 (0.29–14.14)	0.48
	ANC	3.8, 2.8–5.5	1.3, 1.2–2.5	1.93 (0.88–4.22)	0.1
	Mitogen	8.2, 3.7–10	10, 7.9–10	0.91 (0.66–1.25)	0.56
	QFM	206, 40–454	164, 36–282	1.02 (0.97–1.08)	0.41

Abbreviations: ALC, absolute lymphocyte count (x1000 cells/µL); ANC, absolute neutrophil count (x1000 cells/µL); QFM, Quantiferon‐Monitor (IU/mL); Mitogen, mitogen component of the QF‐CMV assay (IU/mL).

* Odds ratios displayed per 1 unit decrease in biomarker value except for QFM which is per 10 IU/mL decrease.

**FIGURE 2:**
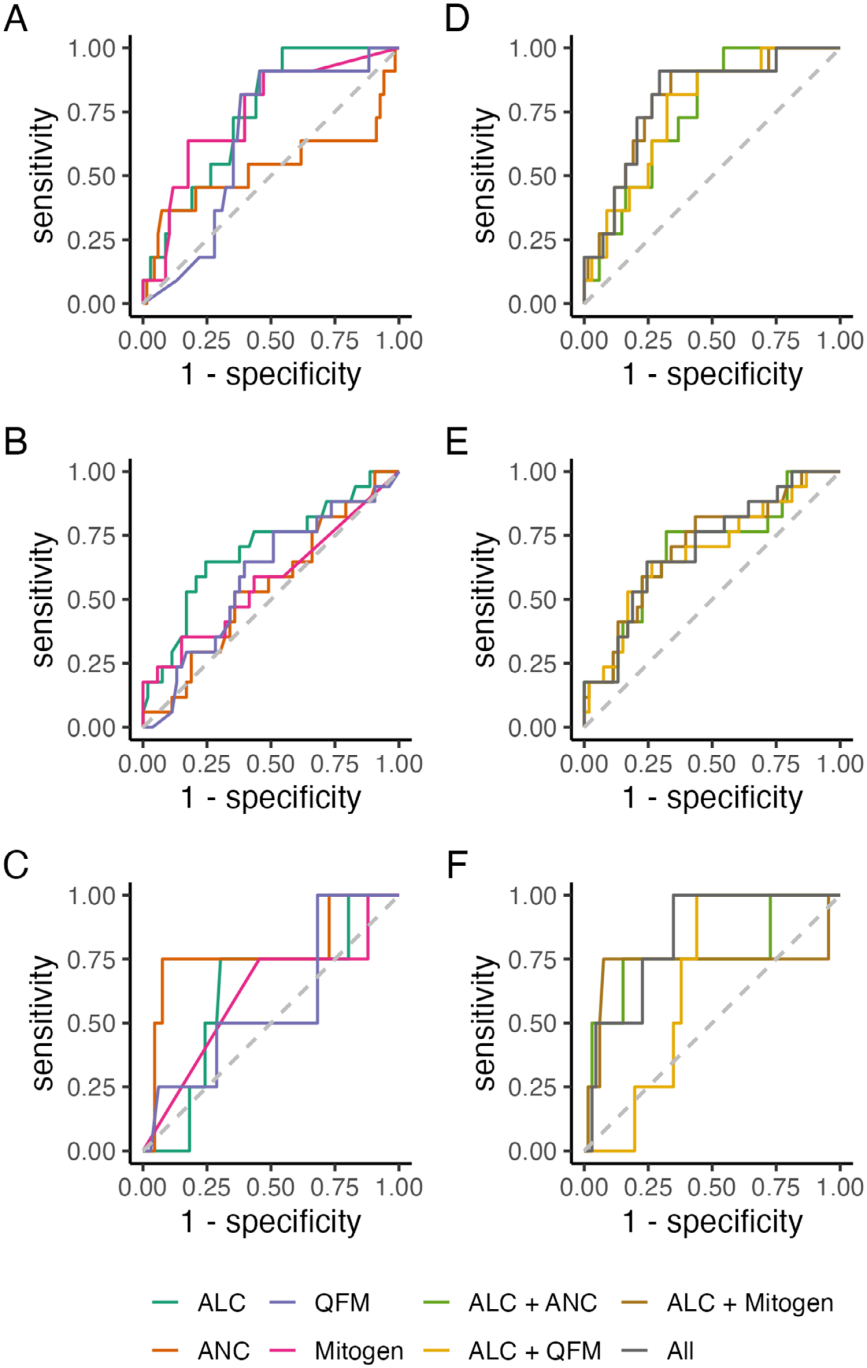
Receiver‐operating characteristic (ROC) curves for predicting serious opportunistic infections with each immune biomarker individually from (a) 3‐6, (b) 6‐12 and (c) 12‐18 months post‐transplant, and multivariate models incorporating several biomarkers from (d) 3‐6, (e) 6‐12 and (f) 12‐18 months post‐transplant. ALC, absolute lymphocyte count. ANC, absolute neutrophil count. QFM, Quantiferon Monitor.

Optimal cutoffs and were calculated using ROC curves (Figure 2). Cutoffs for ALC were relatively stable across all 3 time periods at around 1.0×1000 cells/µL, while for other biomarkers increased over time (Table 2). Individually, the best performing biomarker was ALC particularly early post‐transplant where area under the ROC curves (AUC) was 75% and 70% respectively. Sensitivity, specificity, positive & negative predictive values for an ALC threshold of 1.1×1000 cells/µL were 73%, 65%, 25% & 94% between 3–6 months, and for a threshold of 1.0×1000 cells/ µL were 65%, 76%, 46% & 87% from 6–12 months respectively. The ANC was poorly predictive in the first year, but between 12–18 months predictive accuracy increased with an AUC of 78%. Overall, negative predictive values were consistently high, meaning that patients with higher biomarker values were unlikely to develop infections.

**TABLE 2:** Performance characteristics for the four immune biomarkers alone and in combination for each time period.

Time period	Model	Optimal cutoff	No. (%) patients testing below cutoff	Specificity (%)	Sensitivity (%)	PPV (%)	NPV (%)	AIC	AUC (%)
3–6 months	ALC	1.1	47 (60%)	65%	73%	25%	94%	59.7	75% (61%–88%)
	ANC	2.9	19 (24%)	79%	46%	26%	90%	67.7	53% (28%–78%)
	Mitogen	0.46	60 (76%)	82%	64%	37%	93%	60.6	74% (58%–90%)
	QFM	10	44 (56%)	62%	82%	26%	96%	63.5	64% (50%–79%)
	ALC + ANC	—	54 (68%)	74%	64%	28%	93%	61.6	75% (62%–88%)
	ALC + mitogen	—	52 (66%)	74%	82%	33%	96%	57.7	80% (66%–93%)
	ALC + QFM	—	48 (61%)	68%	82%	29%	96%	60.7	76% (62%–89%)
	All	—	49 (62%)	71%	91%	33%	98%	61.6	80% (66%–94%)
6–12 months	ALC	1.0	46 (66%)	76%	65%	46%	87%	75.4	70% (54%–85%)
	ANC^*^	5.3	42 (60%)	64%	53%	32%	81%	81.3	55% (39%–70%)
	Mitogen	8.4	37 (53%)	57%	59%	30%	81%	80.8	57% (40%–74%)
	QFM	60	38 (54%)	60%	65%	34%	84%	81.0	59% (43%–74%)
	ALC + ANC	—	40 (57%)	68%	77%	43%	90%	76.8	69% (54%–84%)
	ALC + mitogen	—	40 (57%)	66%	71%	40%	88%	76.8	71% (56%–85%)
	ALC + QFM	—	45 (64%)	74%	65%	44%	87%	77.3	69% (53%–84%)
	All	—	46 (66%)	76%	65%	46%	87%	80.1	70% (56%–85%)
12–18 months	ALC	1.0	47 (67%)	70%	75%	13%	98%	34.1	62% (33%–91%)
	ANC	1.5	62 (89%)	92%	75%	38%	98%	30.8	78% (44%–100%)
	Mitogen^*^	9.38	37 (53%)	55%	75%	9%	97%	34.3	61% (29%–93%)
	QFM	56	49 (70%)	71%	50%	10%	96%	33.8	58% (26%–89%)
	ALC + ANC	—	57 (81%)	85%	75%	23%	98%	32.4	77% (44%–100%)
	ALC + mitogen	—	62 (89%)	92%	75%	38%	98%	35.4	73% (28%–100%)
	ALC + QFM	—	37 (53%)	56%	100%	12%	100%	35.5	66% (52%–80%)
	All	—	52 (74%)	77%	75%	17%	98%	34.2	84% (68%–100%)

Abbreviations: ALC, absolute lymphocyte count (x1000 cells/µL); ANC, absolute neutrophil count (x1000 cells/µL); QFM, Quantiferon‐Monitor (IU/mL); Mitogen, mitogen component of the QF‐CMV assay (IU/mL); PPV, positive predictive value; NPV, negative predictive value; AIC, Akaike information criterion; AUC, area under the receiver operating characteristic curve.

* Values refer to patients testing below the calculated optimal cutoffs relative to those testing above, except for the asterisked models where the interpretation is reversed (likely due to poor discrimination).

Combining multiple biomarkers together did modestly improve model performance compared to each individual biomarker alone (Table 2). The combination of biomarkers that performed best varied over time. Between 3–6 months post‐transplant, adding mitogen values to ALC increased the AUC from 75% to 80%. From 6–12 months, combinations were no better than ALC alone. After 12 months, adding ANC to ALC increased AUC from 62% to 77%, although using all 4 biomarkers further improved it to 84%.

## 4 Discussion

Lung transplant recipients experience a high burden of infections which contribute to morbidity and mortality. Immune biomarkers, despite offering the potential to measure the net state of immunosuppression and identify patients at higher risk for serious infections, have not yet been implemented into routine practice. It is not clear whether a pathogen‐specific or a pathogen‐agnostic approach is best. In this study, we evaluated four global immune biomarkers, alone and in combination, as predictors of a clinically meaningful outcome, hospitalization from infections related to immunosuppression (SOIs). The ALC was the single most useful test, although incorporating 3 other biomarkers modestly improved predictions, with the utility of each varying over time. Negative predictive values were high, indicating that patients with high biomarker values were less likely to experience SOI.

Multiple different biomarkers have been evaluated in transplant recipients previously, with variable performance. ALC, a simple, inexpensive, readily & reliably measurable value, is increasingly associated in multiple studies with both CMV and other opportunistic infections. [8, 11, 12, 13, 14, 30, 31] Our prior study demonstrated the potential for QFM to identify overly immunosuppressed patients at risk for SOI from 3–12 months post‐transplant, findings which have also been seen by others. [9, 32] Here, while patients with SOI did have lower QFM values, our analyses did not reach statistical significance, likely due to a different analytic approach and fewer outcomes occurring beyond 12 months. ANC was not predictive of SOI early post‐transplant, although interestingly performed well at 12 months. Low ANC values often prompt modifications to therapy such as reduction in mycophenolate/azathioprine or withdrawal of valganciclovir/cotrimoxazole, which could have impacted subsequent infection risk. While not specifically developed as a stand‐alone test, the mitogen component of the Quantiferon‐CMV assay, which is also included in the Quantiferon‐TB assay, is available. There are several previous studies that have found that indeterminate results and low mitogen values are associated with a high risk of CMV. [15, 16, 33, 34]

The complexity of the immunological response makes it unlikely that any single marker will be highly predictive of clinical risks associated with immunosuppression. The combined use of distinct biomarkers may offer more accurate predictions than the use of single markers alone. [35] Several immunological scores incorporating multiple biomarkers and clinical factors have been developed and validated to try and improve predictions and identify patients at higher risk of CMV infection, but none have widespread uptake thus far [36, 37, 38, 39, 40, 41, 42, 43, 44, 45]. These studies have varied in complexity, transplant population assessed, and clinical outcomes evaluated. Amongst 168 kidney transplant recipients, a score to predict infections included mycophenolate use, graft function, CD4+ and NK cell number. [38] In 375 heart transplant recipients a composite infection outcome was predicted by age, diabetes, renal replacement therapy, CMV serostatus, lymphodepletion, trimethoprim‐sulfamethoxazole prophylaxis use, hospitalization and lymphocyte count at 1 month post‐transplant. [13, 42] In 309 kidney and liver recipients, a score to predict late severe infections included EBV DNAemia, age, prior infection, CMV mismatch and CD8+ T‐cell count at 6 months. [43] Finally, a score including age, CD4+ and CD8+ T‐cell counts, C3 level, IgG level and glomerular filtration rate at 1 month was developed in 410 and validated in 522 kidney recipients to predict infections at 6 months. [40]

This study is one of the first to evaluate QFM, mitogen, and ALC/ANC, alone and in combination. All of these tests are commercially available and can be performed in routine diagnostic laboratories, including measures of both cell numbers as well as function. Our findings are novel and clinically relevant, and follow‐up was complete. Our primary outcome, SOI, was specific, precise and rigorously evaluated, focusing on clinically meaningful infections that cause significant morbidity and mortality and are likely to be related to the degree of immunosuppression. Biomarkers were not used for clinical decision making, which could have confounded the relationship with SOI. NPV values were high, meaning that the biomarkers were able to identify patients at low risk for infection. However, there are several limitations to consider when interpreting our results. Statistical power was limited by the available sample size and number of outcomes, particularly between 12–18 months post‐transplant. Blood was collected at defined timepoints only, with 6 months an extended time in which changes could have occurred. Future studies could consider more frequent sampling. A small number of patients had missing data, and we performed a complete case analysis only. Mitogen values are capped at 10 IU/mL, and patients were clustered around this upper limit beyond 6 months, limiting variability which reduced predictive utility. Changing this would require diluting samples or working with the manufacturer to modify the test. There were insufficient patients to understand the contribution of different types of infections (CMV, fungal and others) to the findings. Because all patients received indefinite cotrimoxazole prophylaxis, there were no cases of *Pneumocystis jirovecii* pneumonia in our cohort, limiting the ability to predict this outcome. Thymoglobulin was infrequently used, limiting our ability to analyze this important risk factor for both lymphopenia and infection. It was unlikely to have significantly impacted biomarker results given it was mostly used after the 12‐month blood collection timepoint. We were unable to fully explore the impacts of immunosuppression regimens on immune biomarkers and infections outcomes due to the complex, interrelated and time‐varying nature of these relationships. Finally other outcomes such as rejection, CLAD and mortality were not explored, but could be a focus of future work.

The ability to accurately predict which patients are at risk for serious opportunistic infections after lung transplant would be extremely clinically useful and could inform decision‐making around interventions like immunosuppression dosing, antimicrobial prophylaxis and frequency of clinical/virologic monitoring. For example, most of the SOIs identified in our patients were CMV or fungal infections, and valganciclovir and/or mold‐active azole prophylaxis could easily be initiated in patients with low biomarker values. This would represent an important step forward in an individualized precision‐medicine approach to post‐transplant management. ALC was the single most useful test, and the low cost, simplicity and widespread availability of this is appealing. Other significantly more expensive biomarkers did not outperform ALC. While promising, our findings demonstrate the challenges and complexities of using these assays, which are not yet ready for routine clinical implementation. Larger, carefully designed prospective and interventional studies incorporating these and other novel biomarkers as well as clinical factors are needed to further explore these tests. This will help understand the complex relationships which exist between immunosuppression, immune biomarkers, SOIs and rejection and define how to optimally integrate these assays into practice.

## Author Contributions

Quoc Nguyen and Michael J. Lydeamore performed the analysis and contributed to results interpretation and manuscript writing. Adina Dessauer assisted with data collection. Bradley J. Gardiner was involved in study design, data collection, results interpretation and manuscript preparation. Anton Y. Peleg, Gregory I. Snell & Glen P. Westall provided supervision, assistance with data interpretation and manuscript review. All authors have reviewed the manuscript and agree with its content.

## Funding

BG was supported by a Research Grant from The Transplantation Society, a Research Establishment Fellowship from the Royal Australasian College of Physicians, and a National Health and Medical Research Council (NHMRC) Investigator Grant (2041063). The Lungitude and Sylvia and Charles Viertel Foundations provided study support. Qiagen provided assays free of charge and some research support but had no other input into the study.

## Conflicts of Interest

Qiagen provided the assays free‐of‐charge and provided GW with financial and conference travel support to attend Qiagen Advisory Board meetings but did not have any input into the study design, analysis or manuscript preparation. BG has received funding from Qiagen, Biotest and Takeda. All other authors report no conflicts of interest.

## Supporting information




**Supporting Information**: ctr70654‐sup‐0001‐SuppMat.docx

## Data Availability

The data that support the findings of this study are available on request from the corresponding author. The data are not publicly available due to privacy or ethical restrictions.
