# Supplementary material for: Evaluation of Global Immune Biomarkers to Predict Infections in Lung Transplant Recipients
Source: Clin Transplant. 2026 Aug 17;40(8):e70654. doi: 10.1111/ctr.70654 (PMC13481288; doi:10.1111/ctr.70654)
Supplement: Supplementary file 1 [file CTR-40-e70654-s001.docx]

**Evaluation of Global Immune Biomarkers to Predict Infections in Lung Transplant Recipients**

Quoc Nguyen^1^, Michael J. Lydeamore^1^, Adina Dessauer^2^, Gregory I. Snell^3,4^, Glen P. Westall^3,4^, Anton Y. Peleg^2,4,5,6^, Bradley J. Gardiner^2,4^

^1^Department of Econometrics and Business Statistics, Monash University, Melbourne, Victoria, Australia

^2^Department of Infectious Diseases, Alfred Health, Melbourne, Victoria, Australia

^3^Lung Transplant Service, Department of Respiratory Medicine, Alfred Health, Melbourne, Victoria, Australia

^4^School of Translational Medicine, Monash University, Melbourne, Victoria, Australia

^5^Centre to Impact AMR, Monash University, Clayton, Victoria, Australia

^6^Infection Program, Monash Biomedicine Discovery Institute, Department of Microbiology, Clayton, Victoria, Australia

**Supplementary Figures**

**Figure S1:** Number and flow of study participants.

**Figure S2:** Overall immune biomarker values in the first year (2 & 6 weeks, 3, 6 &12 months) post-transplant including (a) absolute lymphocyte count, x1000 cells/μL (b) absolute neutrophil count, x1000 cells/μL (c) Quantiferon®-Monitor, IU/mL and (d) mitogen values, IU/mL.

**Figure S3:** Kaplan-Meier plot demonstrating time to first serious opportunistic infection within the first 18 months following lung transplant.

**Supplementary Tables**

**Table S1:** Definitions of clinical infection syndromes.

| **Syndrome** | **Details** |
| --- | --- |
| Pneumonia | Appropriate symptoms (productive cough with purulent sputum, shortness of breath, other pulmonary symptoms with or without fever) AND  Presence of pulmonary infiltrate on lung imaging  OR clinical evidence of pulmonary infiltrate and positive microbiology cultures with a consistent organism  AND the absence of acute rejection |
| Bronchitis | Productive cough with purulent sputum OR secretions on bronchoscopy without evidence of lung parenchymal involvement (clinical or imaging) |
| Upper respiratory tract infection | Upper respiratory symptoms (nasal congestion, rhinorrhea etc.) only with or without a positive respiratory viral panel |
| Urinary tract infection | Urinary symptoms (dysuria, frequency etc.) AND pyuria on microscopy with or without positive culture |
| Gastroenteritis | Diarrhea AND fever OR an appropriate pathogen isolated from stool culture |
| Bloodstream infection | Positive blood cultures and attributable febrile illness with no other cause identified |
| Skin/soft tissue including wound infection | Appropriate local symptoms such as erythema, purulent exudate from a wound etc. with or without a positive culture |
| Invasive fungal infection | *Proven:* mold present in tissue biopsy of focal lesion  *Probable:* mold present in BAL and pulmonary nodule/cavity on CT imaging  *Probable without prespecified radiologic criteria:* mold present in BAL and any pulmonary radiologic abnormality  *Possible:* suggestive pulmonary radiologic abnormality but no mold identified or testing not performed |
| Fungal tracheobronchitis | Bronchoscopic abnormality such as necrosis, plaque, eschar, ulceration or pseudomembranes and identification of mold on microbiologic testing |
| Fungal colonization | Absence of symptoms or radiologic/bronchoscopic abnormalities but mold identified on microbiologic/cytologic testing including direct visualization on fungal staining, growth on culture, positive galactomannan or fungal PCR. Mold colonization was not classified as an infection. |
| CMV syndrome | Detection of CMV in blood plus ≥2 of: fever for ≥2 days, new or increased malaise, neutropenia (<1.5 x 10^3^/μL) or thrombocytopenia (<150 x 10^3^/μL), elevated AST or ALT (>2x upper limit of normal). |
| CMV end-organ disease | Laboratory confirmation of CMV plus clinical evidence of organ dysfunction, categorized as *proven* (positive pathology at a non-blood site with attributable symptoms), *probable* (DNAemia plus attributable symptoms), or *possible* (DNAemia with clinical symptoms suggestive of end-organ involvement but a potential alternative diagnosis present). CMV pneumonitis required evidence of a pulmonary infiltrate on imaging with or without symptoms plus identification of CMV on lung biopsy (proven) or in BAL fluid (probable). |

CMV, cytomegalovirus. BAL, bronchoalveolar lavage.

**Table S2:** Characteristics of the 80 lung transplant recipients in our cohort.

| **Characteristic** | **Details (n=80)** |
| --- | --- |
| Age at transplant (years), median, IQR  Male sex, no. (%)  Underlying disease, no. (%)  COPD  Pulmonary fibrosis  Cystic fibrosis  Pulmonary hypertension  α_1_-antitrypsin deficiency  Other | 61, 57-65  48 (60%)  34 (43%)  24 (30%)  5 (6%)  3 (4%)  4 (5%)  10 (12%) |
| Pre-transplant comorbidities, no. (%)  Hypertension  Diabetes  Underlying cardiac disease  Previous thoracic surgery  Prednisolone use  Other immunosuppression  Intravenous immune globulin use | 21 (26%)  8 (10%)  21 (26%)  8 (10%)  32 (40%)  3 (4%)  7 (9%) |
| Transplant details  Bilateral (vs. single) lung transplant, no. (%)  Average ischemic time (mins), mean ± SD  Surgery duration (mins), median, IQR  Cardiopulmonary bypass, no. (%)  ECMO, no. (%)  Time intubated (hours), median, IQR  Reintubated, no. (%)  Initial ICU LOS (days), median, IQR  ICU readmission, no. (%)  Hospital LOS (days), median, IQR | 69 (86%)  284 ± 105  236, 103-656  10 (12%)  7 (9%)  22, 15-48  10 (12%)  4, 3-8  10 (12%)  19, 16-28 |
| CMV serostatus, no. (%)  D+/R-  D+/R+  D-/R+  D-/R- | 20 (25%)  32 (40%)  19 (24%)  9 (11%) |
| EBV serostatus, no. (%)  D+/R-  D+/R+  D-/R+ | 3 (4%)  74 (93%)  3 (4%) |
| Immunosuppression, no. (%)  Basiliximab induction  Azathioprine (vs mycophenolate) | 40 (50%)  63 (79%) |
| Outcomes (within 18 months), no. (%)  Any infection  Serious infection  Opportunistic infection  Serious opportunistic infection  Rejection  Death | 78 (98%)  63 (79%)  55 (69%)  35 (44%)  25 (31%)  6 (8%) |

IQR, interquartile range. COPD, chronic obstructive pulmonary disease. SD, standard deviation. ECMO, extracorporeal membrane oxygenation. ICU, intensive care unit. LOS, length of stay. CMV, cytomegalovirus. D, donor. R, recipient. EBV, Epstein-Barr virus.

**Table S3:** Immune biomarker results (median, IQR), total number and proportion of patients remaining alive with results available from each timepoint post-transplant.

| **Biomarker**  No. patients | **2 weeks**  79 (99%) | **6 weeks**  79 (99%) | **3 months**  79 (99%) | **6 months**  78 (98%) | **12 months**  75 (94%) |
| --- | --- | --- | --- | --- | --- |
| ALC | 1.1, 0.8-1.6  79 (99%) | 1.2, 0.8-1.7  78 (98%) | 1.2, 0.9-1.8  79 (99%) | 1.3, 0.8-1.8  70 (88%) | 1.2, 0.9-1.6  70 (88%) |
| ANC | 8.5, 6.2-10.9  79 (99%) | 5.8, 4.5-7.2  78 (98%) | 4.3, 3.0-6.1  79 (99%) | 4.5, 2.9-6.5  70 (88%) | 3.7, 2.6-5.4  70 (88%) |
| Mitogen | 0.3, 0.1-1.4  79 (99%) | 1.7, 0.3-5.8  78 (98%) | 4.3, 0.5-10  78 (98%) | 8.5, 1.9-10  77 (96%) | 8.2, 3.6-10  75 (94%) |
| QFM | 3, 1-8  79 (99%) | 8, 3-47  78 (98%) | 28, 2-150  79 (99%) | 73, 14-208  77 (96%) | 178, 36-440  75 (94%) |

ALC, absolute lymphocyte count (x1000 cells/μL). ANC, absolute neutrophil count (x1000 cells/μL). IQR, interquartile range. QFM, Quantiferon®-Monitor (IU/mL). Mitogen, mitogen component of the QF-CMV assay (IU/mL).

**Table S4:** Summary of serious opportunistic infection (SOI) outcomes by time period post-transplant.

| **Months post-transplant** | **No. patients with SOI (%*)** | **Total no. SOIs** | **SOI details** |
| --- | --- | --- | --- |
| 0-3 | 7/79 (9%) | 9 | 7 invasive fungal (4 yeast, 3 mold), 1 *Nocardia* sp., 1 *M. abscessus* |
| 3-6 | 12/79 (15%) | 13 | 9 invasive fungal (7 mold, 1 *Cryptococcus*, 1 *Candida* sp.), 3 CMV, 1 VZV |
| 6-12 | 18/75 (24%) | 19 | 9 CMV, 8 invasive fungal (4 mold, 2 *Cryptococcus*, 2 *Candida* sp.), 1 *Nocardia*, 1 *M. abscessus* |
| 12-18 | 4/74 (5%) | 5 | 2 CMV, 1 invasive fungal (*Cryptococcus*), 1 *M. abscessus*, 1 EBV |

*as a proportion of the number of patients that survived to the end of each time period

SOI, serious opportunistic infection. CMV, cytomegalovirus. VZV, varicella zoster virus. EBV, Epstein Barr virus.

**Table S5:** Description of the 46 individual episodes of serious opportunistic infection.

| **Time post-transplant** | **Patient** | **Day of onset** | **Duration of hospitalization (days)** | **Clinical syndrome** | **Pathogen** |
| --- | --- | --- | --- | --- | --- |
| <2 weeks | 6 | 11 | 4 | Probable pulmonary IFI without typical radiology | *Aspergillus fumigatus* |
|  | 39 | 1 | * | Candidemia | *Candida albicans* |
| 2-6 weeks | 4 | 21 | * | Empyema | *Candida albicans* |
|  | 6 | 30 | 5 | Pneumonia | *Nocardia nova* |
|  | 10 | 33 | * | Probable pulmonary IFI without typical radiology | *Aspergillus fumigatus* |
|  | 15 | 39 | 5 | Empyema | *Candida dubliniensis* |
|  | 74 | 22 | * | Esophageal candidiasis | *Candida sp.* |
| 6 weeks – 3 months | 28 | 45 | 25 | Pneumonia | *Mycobacterium abscessus* |
|  | 39 | 77 | 12 | Tracheobronchitis | *Aspergillus sp.* |
| 3-6 months | 8 | 176 | 12 | Probable pulmonary IFI without typical radiology | *Fusarium verticillioides* |
|  | 12 | 86 | 1 | Esophageal candidiasis | *Candida albicans* |
|  | 14 | 186 | 5 | Cytomegalovirus syndrome | Cytomegalovirus |
|  | 22 | 155 | 21 | Probable pulmonary IFI without typical radiology | *Aspergillus sp.* |
|  | 23 | 186 | 13 | Probable gastrointestinal, possible pneumonitis | Cytomegalovirus |
|  | 24 | 160 | 57 | Probable pulmonary IFI without typical radiology | *Rhizomucor miehei* |
|  | 25 | 154 | 17 | Proven pulmonary IFI | *Aspergillus fumigatus* |
|  | 25 | 220 | 15 | Probable gastrointestinal disease | Cytomegalovirus |
|  | 26 | 200 | 3 | Pulmonary cryptococcosis | *Cryptococcus neoformans* |
|  | 37 | 176 | 18 | Probable pulmonary IFI | *Scedosporium apiospermum* |
|  | 38 | 163 | 5 | Probable pulmonary IFI | *Aspergillus sp.* |
|  | 62 | 103 | * | Disseminated zoster | Varicella zoster virus |
|  | 71 | 161 | 7 | Probable pulmonary IFI without typical radiology | *Aspergillus fumigatus* |
| 6-12 months | 1 | 238 | 10 | Probable pneumonitis | Cytomegalovirus |
|  | 3 | 383 | 5 | Probable pneumonitis | Cytomegalovirus |
|  | 7 | 235 | 1 | Asymptomatic | Cytomegalovirus |
|  | 10 | 290 | 1 | Probable pulmonary IFI without typical radiology | *Aspergillus nidulans* |
|  | 11 | 362 | 3 | Pulmonary cryptococcosis | *Cryptococcus neoformans* |
|  | 17 | 359 | 34 | Probable pulmonary IFI without typical radiology | *Aspergillus sp.* |
|  | 18 | 250 | 3 | Lung, pleura, rib osteomyelitis (incision) | *Mycobacterium abscessus* |
|  | 22 | 361 | 5 | Probable pulmonary IFI | *Aspergillus sp.* |
|  | 30 | 216 | 5 | Breast abscess | *Candida dubliniensis* |
|  | 35 | 207 | 7 | Probable pulmonary IFI | *Lomentospora prolificans* |
|  | 35 | 384 | 13 | Asymptomatic | Cytomegalovirus |
|  | 36 | 366 | 12 | Probable gastrointestinal disease | Cytomegalovirus |
|  | 37 | 214 | 18 | Asymptomatic | Cytomegalovirus |
|  | 41 | 209 | 7 | Asymptomatic | Cytomegalovirus |
|  | 53 | 391 | 8 | Proven gastrointestinal disease (upper and lower) | Cytomegalovirus |
|  | 54 | 310 | 23 | Pulmonary & bloodstream | *Nocardia* sp. |
|  | 56 | 365 | 2 | Pulmonary cryptococcosis | *Cryptococcus neoformans* |
|  | 66 | 201 | 14 | Asymptomatic | Cytomegalovirus |
|  | 79 | 214 | 5 | Right thoracotomy wound | *Candida albicans* |
| 12-18 months | 10 | 367 | 5 | Asymptomatic | Cytomegalovirus |
|  | 10 | 375 | 7 | Cytomegalovirus syndrome | Cytomegalovirus |
|  | 18 | 544 | 15 | Right chest wall | *Mycobacterium abscessus* |
|  | 36 | 477 | 14 | Viral syndrome (reactivation) | Epstein-Barr virus |
|  | 43 | 414 | 4 | Pulmonary cryptococcosis | *Cryptococcus* sp. |

*occurred during the initial post-transplant hospitalization

IFI, invasive fungal infection.

**Table S6:** Clinical characteristics of lung transplant recipients stratified by serious opportunistic infection status in the first 18 months post-transplant (n=79).

| **Characteristic** | **No serious OI (n=45)** | **Serious OI (n=35)** | **Unadjusted HR (95% CI)** | **p-value** |
| --- | --- | --- | --- | --- |
| Age at transplant (years), median, IQR  Male sex, no. (%)  Underlying disease, no. (%)  COPD  Pulmonary fibrosis  Other | 60, 57-65  29 (64%)  18 (40%)  14 (31%)  13 (29%) | 61, 58-65  19 (54%)  16 (46%)  10 (29%)  9 (26%) | 1.03 (0.98-1.07)  0.77 (0.41-1.46)  Ref  0.83 (0.4-1.74)  0.78 (0.35-1.74) | 0.22  0.42  -  0.62  0.54 |
| Pre-transplant comorbidities, no. (%)  Hypertension  Diabetes  Underlying cardiac disease  Previous thoracic surgery  Prednisolone use  Other immunosuppression  Intravenous immune globulin use | 11 (24%)  6 (13%)  12 (27%)  5 (11%)  20 (44%)  2 (4%)  4 (9%) | 10 (29%)  2 (6%)  9 (26%)  3 (9%)  17 (49%)  2 (6%)  3 (9%) | 1.17 (0.57-2.41)  0.43 (0.10-1.80)  1.12 (0.55-2.25)  0.66 (0.2-2.15)  1.22 (0.65-2.31)  2.23 (0.68-7.25)  0.94 (0.29-3.05) | 0.67  0.25  0.76  0.49  0.54  0.18  0.91 |
| Transplant details  Bilateral (vs. single) lung transplant, no. (%)  Average ischemic time (mins), mean ± SD  Cardiopulmonary bypass, no. (%)  ECMO, no. (%)  Time intubated (hours), median, IQR  Reintubated, no. (%)  Initial ICU LOS (days), median, IQR  ICU readmission, no. (%)  Hospital LOS (days), median, IQR | 39 (87%)  285 ± 111  6 (13%)  4 (9%)  20 (13-42)  7 (16%)  4 (3-7)  4 (9%)  18 (17-23) | 30 (85%)  283 ± 98  4 (11%)  3 (9%)  26 (21-50)  3 (9%)  4 (3-8)  6 (17%)  21 (16-38) | 0.67 (0.28-1.61)  1.00 (1.00-1.00)  1.02 (0.40-2.62)  1.00 (0.31-3.25)  1.00 (1.00-1.00)  0.49 (0.15-1.59)  1.02 (0.97-1.06)  2.10 (0.88-5.04)  1.02 (1.01-1.03) | 0.37  0.40  0.96  1.00  0.28  0.23  0.47  0.10  0.002 |
| CMV serostatus, no. (%)  D-/R-  D-/R+  D+/R-  D+/R+ | 7 (16%)  8 (18%)  8 (17%)  22 (49%) | 2 (6%)  11 (31%)  12 (34%)  10 (29%) | Ref  3.85 (0.85-18.42)  4.23 (0.95-18.79)  1.93 (0.43-8.62) | -  0.08  0.06  0.39 |
| EBV serostatus, no. (%)  D-/R+  D+/R-  D+/R+ | 1 (2%)  1 (2%)  43 (96%) | 2 (6%)  2 (6%)  31 (87%) | Ref  0.95 (0.13-6.74)  0.44 (0.10-1.83) | -  0.96  0.26 |
| Immunosuppression, no. (%)  Basiliximab induction  Azathioprine (vs mycophenolate) | 24 (53%)  39 (87%) | 16 (46%)  24 (69%) | 0.74 (0.39-1.40)  0.55 (0.24-1.25) | 0.34  0.16 |
| Rejection | 14 (31%) | 11 (31%) | 1.36 (0.70-2.63) | 0.36 |

OI, opportunistic infection. HR, hazard ratio. IQR, interquartile range. COPD, chronic obstructive pulmonary disease. SD, standard deviation. ECMO, extracorporeal membrane oxygenation. ICU, intensive care unit. LOS, length of stay. CMV, cytomegalovirus. D, donor. R, recipient. EBV, Epstein-Barr virus.

**Table S7:** Multivariate odds ratios for serious opportunistic infection between 3-6, 6-12 and 12-18 months post-transplant.

| **Months post-transplant** | **Biomarker** | **Adjusted OR***  **(95% CI)** | **p-value** |
| --- | --- | --- | --- |
| 3-6 | ALC | 3.86 (0.95-15.7) | 0.06 |
|  | ANC | 1.02 (0.81-1.28) | 0.88 |
|  | Mitogen | 1.24 (0.95-1.63) | 0.12 |
|  | QFM | 0.99 (0.88-1.11) | 0.86 |
| 6-12 | ALC | 3.44 (1.19-10.01) | 0.02 |
|  | ANC | 0.92 (0.77-1.11) | 0.4 |
|  | Mitogen | 1.07 (0.91-1.26) | 0.42 |
|  | QFM | 1.00 (0.97-1.02) | 0.93 |
| 12-18 | ALC | 3.89 (0.25-59.63) | 0.33 |
|  | ANC | 1.81 (0.8-4.07) | 0.15 |
|  | Mitogen | 0.77 (0.51-1.17) | 0.22 |
|  | QFM | 1.05 (0.96-1.15) | 0.25 |

*Odds ratios displayed per 1 unit decrease in biomarker value except for QFM which is per 10 IU/mL decrease.

ALC, absolute lymphocyte count (x1000 cells/μL). ANC, absolute neutrophil count (x1000 cells/μL). QFM, Quantiferon®-Monitor (IU/mL). Mitogen, mitogen component of the QF-CMV assay (IU/mL).
